# Impact of Tenofovir Disoproxil‐Induced Fanconi Syndrome on Bone Material Quality: A Case Report

**DOI:** 10.1002/jbm4.10506

**Published:** 2021-05-25

**Authors:** Benjamin Hadzimuratovic, Judith Haschka, Markus A Hartmann, Stéphane Blouin, Nadja Fratzl‐Zelman, Jochen Zwerina, Roland Kocijan

**Affiliations:** ^1^ Ludwig Boltzmann Institute of Osteology at Hanusch Hospital of OEGK and AUVA Trauma Centre Meidling Vienna Austria; ^2^ First Medical Department Hanusch Hospital Vienna Austria; ^3^ Sigmund Freud University Medical Faculty of Bone Diseases Vienna Austria

**Keywords:** BONE BIOPSY, HYPOPHOSPHATEMIA, OSTEOMALACIA, TENOFOVIR DISOPROXIL

## Abstract

Tenofovir is a nucleotide analog reverse‐transcriptase inhibitor (NtARTI) used for treatment of chronic hepatitis B and human immunodeficiency virus (HIV). Fanconi syndrome (FS) is a condition affecting the proximal tubules of the kidney, leading to increased passage and impaired reabsorption of various small molecules such as glucose, phosphate, bicarbonate, and amino acids. Tenofovir disoproxil fumarate (TDF) is one of two pro‐drugs of tenofovir associated with a greater nephrotoxicity and renal complications such as FS with subsequent osteomalacia, acute kidney injury, and reduction of glomerular filtration rate (GFR) compared with tenofovir alafenamide (TAF). We present the case of a 33‐year‐old white woman treated with TDF because of chronic hepatitis B infection suffering four atraumatic fractures over the period of 2 years. The patient was taken off the TDF regimen 3 months before presentation. Initial blood and urine samples suggested the presence of TDF‐induced osteomalacia, which was confirmed by transiliac bone biopsy and histomorphometry. Moreover, bone mineral density distribution (BMDD) by quantitative backscattered electron imaging (qBEI) analysis showed that approximately 56% of the bone surface was normally mineralized and 44% showed a reduced mineralization consistent with the presence of osteomalacia. The patient made a significant recovery upon cessation of the causative agent. This case report emphasizes the use of bone biopsy, histomorphometry and qBEI in confirming the diagnosis of drug‐induced Fanconi syndrome and associated osteomalacia. © 2021 The Authors. *JBMR Plus* published by Wiley Periodicals LLC on behalf of American Society for Bone and Mineral Research.

## 1 Introduction

Tenofovir disoproxil (TDF)‐induced Fanconi syndrome (FS) is a rare but severe complication in hepatitis B and HIV patients, leading to osteomalacia, pain, multiple fractures, and delayed fracture healing. Antiresorptive agents including bisphosphonates and denosumab even worsen the bone quality and clinical outcome.^(^ ^1^ ^)^ Although TDF‐induced osteomalacia can be reflected by low phosphate and high alkaline phosphatase in serum, bone biopsy assures the diagnosis and gives information on the magnitude of bone deterioration. Since information on bone biopsy findings in TDF‐induced osteomalacia is scarce, we accurately investigated the bone sample of an affected female patient including bone histomorphometry and quantitative backscattered electron imaging (qBEI) analyses.

## 2 Case Report

A 33‐year‐old white woman was referred to our outpatient clinic after suffering four atraumatic fractures between the age of 30 and 32 years. The fractures included a spontaneous fracture of the third right metatarsal bone with a spontaneous refracture 2 months later, a longitudinal fracture of the left tibia with protracted healing, as well as a fracture of the right femoral neck treated surgically, likewise with protracted healing. The patient reported contracting a hepatitis B virus infection in her childhood from a blood transfusion after a spinal surgery for neuroblastoma. Consequently, she has been treated with tenofovir disoproxil over the course of 15 years. Furthermore, she also received a course of radio‐ and chemotherapy as a part of the treatment. In addition, medical history revealed an early colon cancer at the age of 25 years, which was treated by surgery and chemotherapy. A genetic testing for most common genetic causes of breast and colon cancer was declined by the patient. Laboratory records showed low iron levels, hypophosphatemia, and hypouricemia, as well as a high alkaline phosphatase. Further blood and urine tests performed at 2 and 3 months before admission in our institution also revealed glycosuria and proteinuria with decreased bicarbonate levels in presence of normal blood glucose levels (Table 1). One year before the first presentation at our institute, the patient received oral vitamin D (cholecalciferol) and calcium carbonate substitution as well as one cycle of denosumab treatment, due to the sustained fractures. The antiviral therapy was stopped 3 months before the first presentation in our outpatient clinic and an oral phosphate substitution was then started.

**Table 1:** Patient's Blood and Urine Sample Findings Performed Before Discontinuation of Tenofovir Disoproxil and Referral to Our Osteo‐Endocrinological Outpatient Clinic

Parameter	Laboratory value	Unit	Reference range
Hemoglobin	12.80	g/dL	12.0–16.0
Platelets	278	10^9^/L	150–350
White blood cell count	5.92	10^9^/L	4.00–10.00
C‐reactive protein	0.06	mg/L	<5.00
**Iron**	**20.00**	**μg/dL**	**35.00–105.00**
Potassium	3.79	mmol/L	3.50–5.10
Sodium	142	mmol/L	136–145
Calcium	2.20	mmol/L	2.15–2.50
**Phosphate**	**0.69**	**mmol/L**	**0.81–1.45**
Magnesium	0.96	mmol/L	0.66–1.07
**Creatinine**	**1.02**	**mg/dL**	**0.50–0.90**
**Uric acid**	**1.60**	**mg/dL**	**2.40–5.70**
**Alkaline phosphatase**	**279**	**U/L**	**35–105**
**Bicarbonate**	**18.40**	**mmol/L**	**21.00–26.00**
**Glucose**	**97**	**mg/dL**	**74–109**
**Protein (urine)**	**300**	**mg/dL**	**Semiquantitative**
**Glucose (urine)**	**75**	**mg/dL**	**Semiquantitative**

All of the values in Table 1 written in bold letters represent blood or urine values which were not within the normal reference range for the respective test. In terms of protein and glucose levels in urine, both of these were elevated in a semiquantitative urine dipstick test hence a normal reference range was not stated. When compared to the chart, protein level of 300 mg/dl in the urine dipstick test would represent a Grade +++ proteinuria, whereas a glucose level of 75mg/dl in the same test would represent a weakly positive finding i.e. Grade defined as trace finding. All of the other values represent serum values and the reference ranges were stated accordingly.

The patient's mother had also sustained a femoral fracture in the past. Two female siblings aged 25 and 35 years, as well as her 3‐year‐old boy were healthy. The family history also included cases of breast cancer in the family, with patient's mother, aunt, and grandmother being affected. The patient negated having spontaneous fractures or tooth loss during her childhood. Nicotine, alcohol, and substance abuse were ruled out. The patient reported having regular menstrual cycle and regular gynecologic checks, which did not yield any pathological findings.

### 2.1 Clinical examinations

The blood analysis performed at admission to our outpatient clinic revealed a normal blood phosphate level due to the ongoing oral phosphate substitution and after stopping the tenofovir regimen 3 months before the presentation. The patient's renal function was normal (creatinine 0.93 mg/dL [0.51–0.95]), uric acid levels were lowered (2.2 mg/dL [2.6–6.0]), while alkaline phosphatase level was markedly elevated (235 U/l [30–120]). The parathyroid hormone was within normal range (69 pg/mL [12–88]), FGF‐23 levels were decreased (10.9 pg/mL [23.2–95.4]), and calcitriol was slightly above the range of normal (99.0 pg/mL [19.9–79.3]). The patient's urine sample revealed elevated levels of total protein (70 mg/dL [0–29]) and glucose (100 mg/dL [0–15]). A subsequent quantitative analysis of patient's urine showed decreased specific gravity (1011 kg/L [1012–1030]) in presence of an increased total protein to creatinine ratio (660 mg/g [0–149]). The patient presented with normal level of cholecalciferol (25‐OH‐vitamin D, 120 nmol/L [75–250]) since she was receiving an adequate substitution that was continued up to the transiliac bone biopsy, which took place 1 month after the presentation at our department. Furthermore, a single course of glucose 1‐phosphate substitution was initiated 2 months before the admission. The patient received tetracycline labeling before biopsy to assess the dynamic bone formation. The patient also received a course of calcitriol (1,25‐vitamin D), which was discontinued due to low tolerance. However, the patient's serum calcitriol levels remained within the normal range even after the cessation of the substitution. Upon acquisition of the transiliac bone biopsy results, the patient received an oral phosphate substitution with 6.4 mmol two times daily as well as an oral cholecalciferol substitution. The phosphate substitution was augmented by additional 6.4 mmol per day. Regular blood checkups showed normal levels of 25‐OH‐vitamin D and 1,25‐vitamin D over the course of the treatment.

Bone scintigraphy showed an increased tracer uptake at the fracture sites, without any signs of malignant process. Bone mineral density (BMD) measurement by dual energy x‐ray absorptiometry (DXA) revealed only osteopenic *T*‐scores at the lumbar spine (L_1_ to L_4_: −1.6) and hip (femoral neck: −1.3, total hip −1.5). High‐resolution peripheral quantitative computed tomography (HR‐pQCT) showed a severe trabecular structural defect at the distal radius and even more at the tibia in presence of normal cortical thickness (Table 2). The examination of oral cavity upon admission in the outpatient clinic yielded a partial anodontia (five teeth), periodontal disease as well as reduction of the osseous substance of the jaw. The patient underwent genetic testing because hereditary osteomalacia, osteogenesis imperfecta, and osteoporosis were initially suspected as the possible cause of multiple fractures. No pathological gene sequences were found in following genes: COL1A2, IFITM5, LEPRE1, PLS3, LRP5, WNT3A, WNT1, DMP1, PHEX, CLCN5, ENPP1, FGF23, SLC34A1, and ALPL. The only pathological finding was the presence of the Sp1‐Polymorphism of the COL1A1 gene in a heterozygotic state. A physical examination revealed moderate swelling of the left forefoot with presence of tenderness and incalescence. Furthermore, the patient showed a limping gait with dragging of the right foot due to suffered fracture of the femur neck.

**Table 2:** Results of High‐Resolution Peripheral Quantitative Computed Tomography (HR‐pQCT)

Parameter	Left radius	Radius—median value for controls (interquartile range)	Right tibia	Tibia—median value for controls (interquartile range)
Total vBMD (HA/cm^3^)	337.5	325.7 (291.4, 386.3)	237.5	305.3 (270.2, 347.3)
Cortical vBMD (HA/cm^3^)	930.0	879.5 (849.5, 903.4)	885.5	874.9 (832.0, 902.7)
Cortical thickness	0.88	0.775 (0.685, 0.878)	1.10	1.130 (0.990, 1.410)
Trabecular density (HA/cm^3^)	109.4	160.4 (149.2, 190.0)	91.7	169.3 (155.0, 200.7)
Trabecular bone volume	0.091	0.129 (0.122, 0.158)	0.076	0.141 (0.130, 0.170)
Trabecular number (mm^−1^)	1.41	1.92 (1.76, 2.23)	1.16	1.76 (1.59, 2.08)
Trabecular thickness (mm)	0.065	0.075 (0.065, 0.086)	0.066	0.081 (0.074, 0.087)
Inhomogeneity (Tb.1/N.SD, mm)	0.326	0.186 (0.158, 0.210)	0.751	0.221 (0.170, 0.242)

The median values for healthy controls (including interquartile range) were taken from Kocijan and colleagues^(^ ^2^ ^)^.

## 3 Materials and Methods

### 3.1 Bone sample preparation

A transiliac bone biopsy sample was taken under local anesthesia, fixed in 70% ethanol, and dehydrated by an ethanol series and degreased by acetone. Subsequently, the sample was embedded in poly‐methylmethacrylate (PMMA). For bone histomorphometry, 3‐μm‐thin sections were cut from the sample block with a microtome (Leica [Buffalo Grove, IL, USA] SP 2500) and stained by Goldner trichrome. The sections were then visualized in bright field and under polarized light with a light microscope equipped with a digital camera (Zeiss Jena, Germany] Axiophot with an Axiocam HRc). Histomorphometric parameters were evaluated following the guidelines given in Parfitt and colleagues;^(^ ^3^ ^)^ reference values were obtained from Rehman and colleagues.^(^ ^4^ ^)^

### 3.2 Quantitative backscattered electron imaging

The remaining sample block was ground and polished (Logitech [Glasgow, UK] PM5) to obtain a smooth surface. Finally, the surface was carbon coated (AGAR SEM carbon coater) to achieve a conducting surface for measurements in the scanning electron microscope. For more details on sample preparation, see Roschger and colleagues.^(^ ^5^, ^6^ ^)^

qBEI was performed to evaluate bone mineralization density distribution (BMDD) and osteocyte lacunae section (OLS). A Zeiss field emission SEM SUPRA 40 operated at an accelerating voltage of 20 kV and a specimen current of approximately 300 pA was used. The device was calibrated with carbon and aluminium as described in Roschger and colleagues.^(^ ^7^ ^)^ For the BMDD measurement, the entire surface of the biopsy was scanned with a pixel resolution of 1.76 μm. The resulting 8‐bit grayscale images were then converted in weight % calcium (wt.% Ca). The BMDD is the frequency histogram of measured calcium values normalized to 100% bone surface. Consequently, it measures the amount of bone area mineralized with a certain calcium content. BMDD was measured in trabecular and cortical compartment separately. Five different parameters are measured to characterize the obtained BMDD curve:^(^ ^7^ ^)^ (i) CaMean, the mean calcium content; (ii) CaPeak, the most frequent calcium content; (iii) CaWidth, the width of the BMDD at half maximum; (iv) CaLow; and (v) CaHigh measuring the amount of lowly and highly mineralized bone (defined by the 5% and 95% percentiles of healthy adults). Reference data for healthy adults was taken from Hartmann and colleagues (Calcified Tissue International 2021, DOI: 10.1007/s00223‐021‐00832‐5). The analysis of the OLS data was performed on 8‐bit grayscale images with a pixel resolution of 0.9 μm. The lacunae size (area and perimeter), density, and shape (aspect ratio) were obtained as described in Blouin and colleagues and Misof and colleagues.^(^ ^8^, ^9^ ^)^

## 4 Results

### 4.1 Bone histomorphometry

**Fig. 1:**
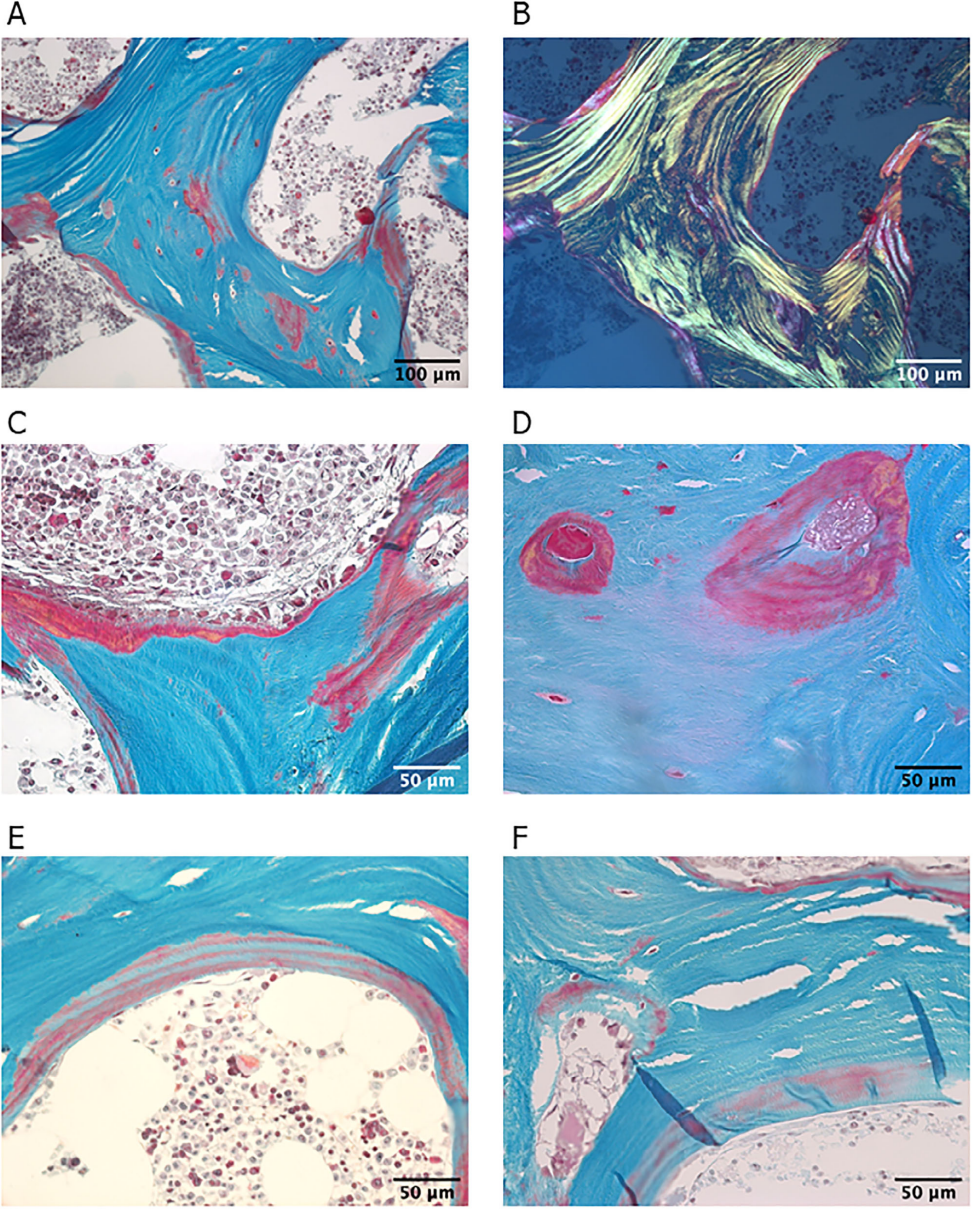
(*A*) Bright‐field light microscopy images of a thin section (3 μm) of trabecular bone stained with Goldner trichrome (green corresponds to mineralized bone matrix, orange/red to non/mineralized bone matrix—osteoid). (*B*) Corresponding polarized light microscopy shows regular lamellar organization. Note the presence of nonmineralized bone tissue in red in the central part of the trabeculae. (*C*–*F*) Bright‐field light microscopy images of higher resolution showing regions with a large amount of osteoid (*C, D*). (*E, F*) Osteoid with interlacing regions stained in green and red, suggesting part mineralization.

The histomorphometric analysis of Goldner‐stained sections showed low cortical thickness (Ct.Wi = 0.60 mm; Z = –2.0), but elevated bone volume per tissue volume (BV/TV = 38.03%, Z = +3.21), due to high trabecular number (Tb.N = 2.88/mm; Z = +2.95), whereas trabecular thickness was within normal range (Tb.Th = 132 μm; Z = –0.73). As shown in Fig. 1, osteoid formation was highly increased with elevated osteoid volume (OV/BV = 15.72%; Z = +12.62), osteoid thickness (O.Th = 17.81 μm; Z = +3.04), and osteoid surface (OS/BS = 58.71%; Z = +11.42). Mineralizing surface per bone surface was increased (MS/BS = 21.34%, Z = +6.34) as well as bone formation rate per bone volume (BFR/BV = 64.09%/y, Z = 2.22). Consistent with these values, mineralization lag time was highly elevated (Mlt = 90.10 days, Z = +8.11). Osteoblast surface was reduced (Ob.S/BS = 1.15%, Z = −2.41), while surface extent of osteoclasts was largely increased (Oc.S/BS = 2.51, Z = +10.05) (Table 3). Polarized light examination showed regular lamellar organization of bone (Fig. 1).

**Table 3:** Results of Bone Histomorphometry and Bone Mineral Density Distribution (BMDD)

Parameter	Patient (*Z*‐score)	Reference values ± SD^a^
Bone histomorphometry		
Ct.Wi (mm)	0.6 (−2.0)	1.15 ± 0.28
BV/TV (%)	38.03 (+3.21)	22.6 ± 4.8
Tb.Th (m)	132.18 (−0.73)	146 ± 19
Tb.N (1/mm)	2.88 (+2.95)	1.7 ± 0.4
OV/BV (%)	15.72 (+12.62)	3.1 ± 1.0
O.Th (μm)	17.81 (+3.04)	8.7 ± 3.0
OS/BS (%)	58.71 (+11.42)	15.3 ± 3.8
Ob.S/BS (%)	1.15 (−2.41)	5.0 ± 1.6
MS/BS (%)	21.34 (+6.34)	7.40 ± 2.20
BFR/BV (%/y)	64.09 (+2.22)	26.1 ± 17.1
Mlt (d)	90.10 (+8.11)	16.3 ± 9.1
Oc.S/BS (%)	2.51 (+10.05)	0.5 ± 0.2
ES/BS (%)	3.49 (−0.51)	4.1 ± 1.2
BMDD of trabecular bone^b^		
CaMean (wt.% Ca)	21.02 (−3.85)	23.26 ± 0.58
CaPeak (wt.% Ca)	23.57 (−1.13)	24.14 ± 0.51
CaWidth (∆wt.% Ca)	7.63 (+13.33)	3.87 ± 0.28
CaLow (% bone area)	19.94 (+9.04)	4.98 ± 1.66
CaHigh (% bone area)	3.36 (−0.47)	4.70 ± 2.84

^a^ Reference values for bone histomorphometry are taken from Rehman and colleagues^(^ ^4^ ^)^.

^b^ For BMDD, from (Hartmann and colleagues, Calcified Tissue International 2021, DOI: 10.1007/s00223‐021‐00832‐5).

### 4.2 Quantitative backscattered electron imaging

**Fig. 2:**
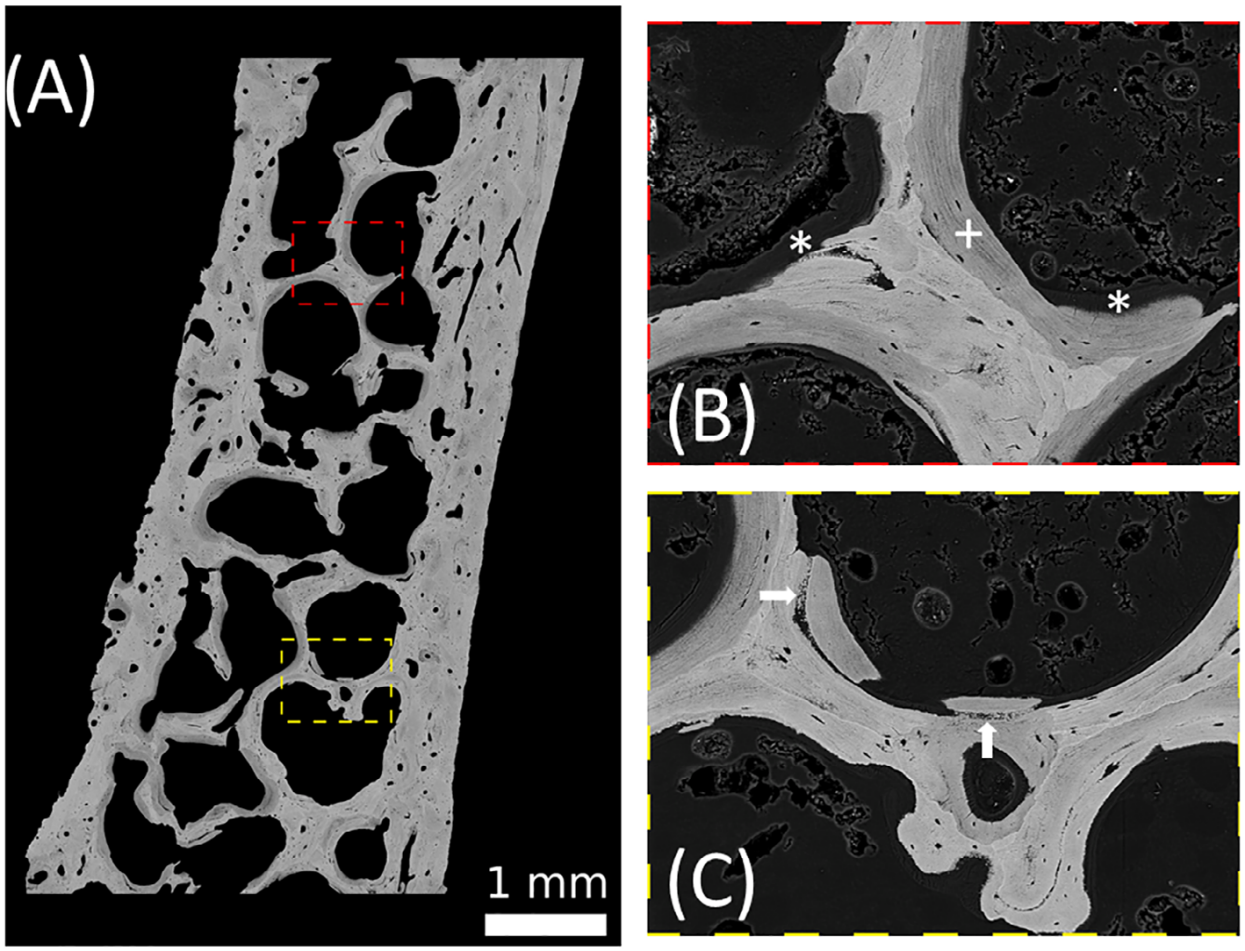
(*A*) Quantitative back‐scattered electron imaging of the transiliac bone biopsy sample. The pixel resolution is 1.7 μm. Brighter gray levels represent areas with a higher calcium content compared with areas with a lower gray level. (*B*, *C*) Images of larger magnification (pixel resolution 0.88 m) of the regions indicated in (*A*). Regions with large amount of osteoid (mineral content smaller than 1 wt.% ca) are marked with an asterisk (*). They are adjacent to lowly mineralized bone packets (as also found in Fig. 2*E*, *F*) indicated with a plus (+). White arrows show regions of abnormal mineralization with isolated mineral particles close to normally mineralized areas.

**Fig. 3:**
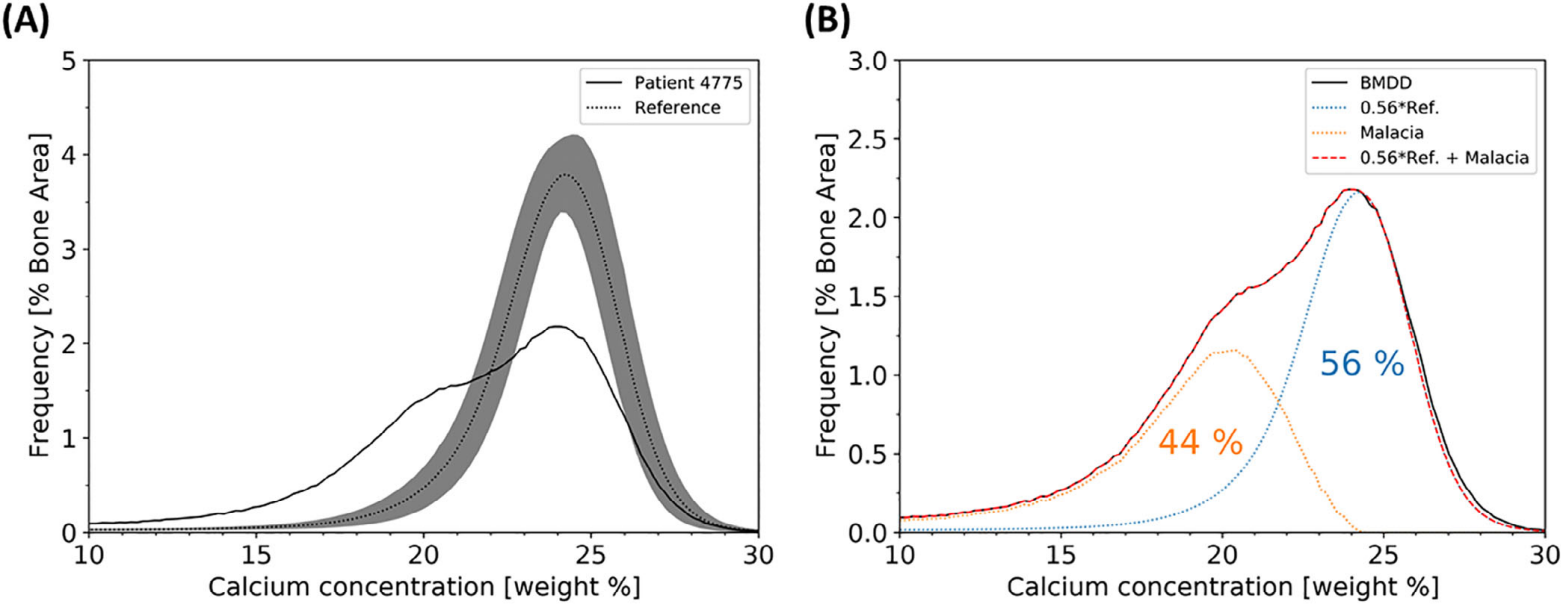
(*A*) The bone mineral density distribution of the patient obtained by qBEI compared with the reference from 25 healthy controls. The curve of the patient shows a double peak compared with the single peak of the reference. (*B*) Demonstration that the patient BMDD curve can be approximated by a sum of a 56% reference curve and an additional curve shifted to low mineralization values due to the numerous low mineralized bone packets opposed to the normal mineralized bone.

Fig. 2*A* shows a backscattered electron image of the biopsy sample. Different gray levels indicate different local mineralization. Two regions in the sample are shown with higher magnification to highlight large amount of osteoid (mineralized less than 1 wt.% Ca) and areas of abnormal mineralization (Fig. 2*B*, *C*). A numerical analysis of the obtained BMDD curve showed that bone matrix mineralization was low in the patient compared with reference values as reflected by decreased values of CaMean (21.02 wt.% Ca, Z = −3.85) and CaPeak (23.57 wt.% Ca, Z = −1.13) and an increase of CaLow (19.94%, Z = +9.04). In contrast, CaHigh (3.36%, Z = −0.47) was not markedly decreased, indicating the presence of lowly and highly mineralized bone matrix (Table 3 and Fig. 3*A*). As a consequence, the heterogeneity in mineralization (CaWidth = 7.63 Δwt.% Ca, Z = +13.33) was increased. Thus, the BMDD of the patient deviates significantly from the reference curve of healthy adults. In particular, there is a broad shoulder at lower mineralization. Fig. 3*B* shows that the shape of the patient's BMDD can be well approximated by the sum of the reference curve and another single peaked curve shifted to lower mineralization. According to this decomposition of the BMDD, approximately 56% of the bone surface are normally mineralized and 44% show a reduced mineralization consistent with the presence of osteomalacia in the patient.

### 4.3 Osteocyte lacunae sections

**Fig. 4:**
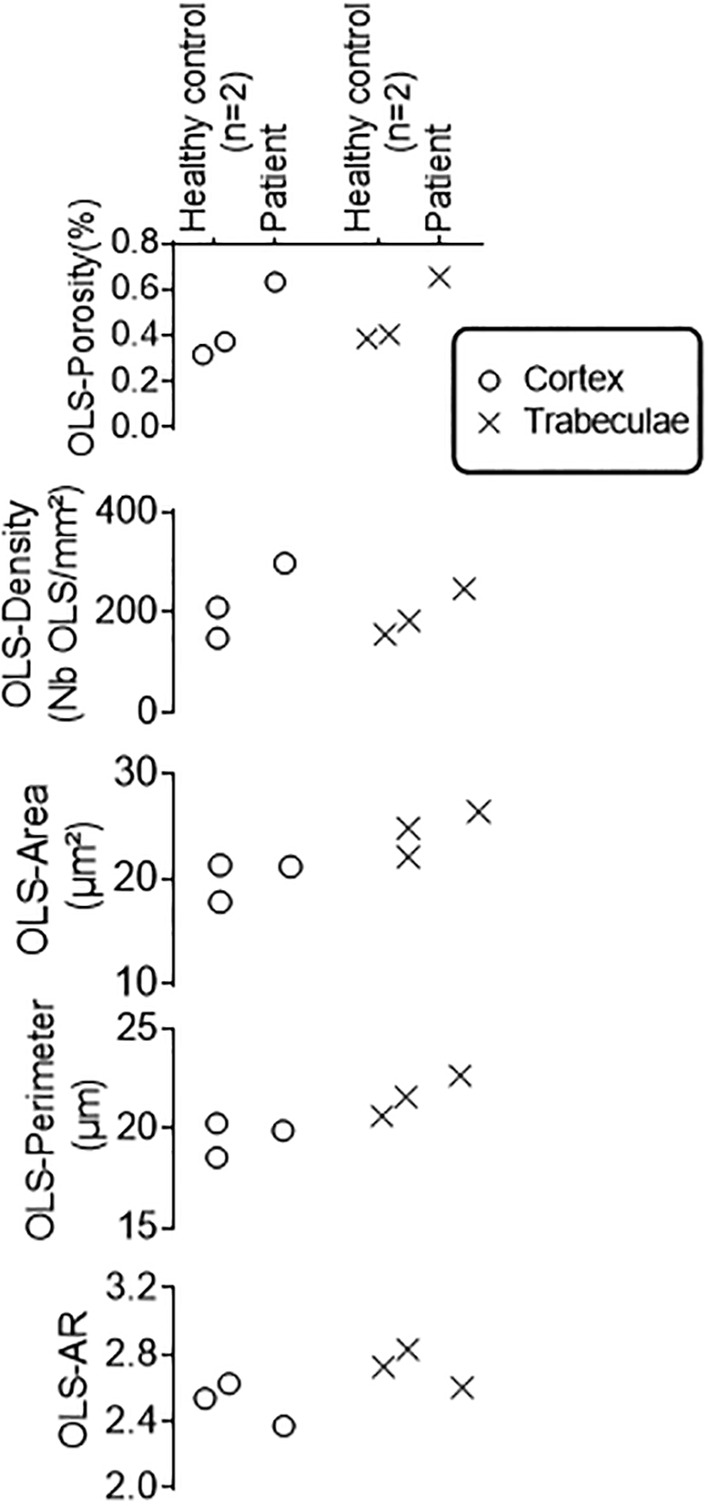
Osteocyte lacunae sections (OLS) data from the patient and two healthy women (36 and 42 years old) obtained from quantitative backscattered electron images with 0.9‐μm pixel resolution. A median bone area of 5.6 mm^2^ (3.2–6.3) and a median OLS number of 868 (634–1504) were measured in cortex and trabecular bone regions, respectively.

Osteocyte lacunae sections (OLS) data (Fig. 4) showed that OLS porosity was elevated in both trabecular and cortical regions. In the trabecular area, OLS were larger compared with the healthy controls^(^ ^10^ ^)^ as observed for OLS‐Area and OLS‐Perimeter parameters. In both compartments, the OLS‐AR was lower compared with control showing a more circular shape of the osteocyte lacunae. The changes in lacunae size and shape can also be found in the Goldner‐stained sections shown in Fig. 1. There the osteocyte lacunae show a red staining characteristic for non‐mineralized bone tissue.

All results summarized led to the diagnosis of an incomplete Fanconi syndrome with marked hypophosphatemic osteomalacia.

## 5 Discussion

We report extensively on the bone biopsy findings of a patient with incomplete Fanconi syndrome and severe osteomalacia, resulting in multiple atraumatic fractures due to longstanding TDF treatment. The 33‐year‐old woman received TDF, a nucleotide reverse transcriptase inhibitor, for the treatment of chronic hepatitis B infection over a course of 15 years.

TDF is known to negatively impact bone metabolism; however, the causes are multifactorial. For once, a greater BMD loss compared with non‐TDF users and an increase in bone turnover markers have been shown in TDF‐treated HIV patients.^(^ ^11^ ^)^ Moreover, TDF can rarely cause acquired Fanconi syndrome, characterized by a proximal tubular dysfunction, wasting of phosphate, and thereby hypophosphatemia, osteomalacia, and muscle weakness.^(^ ^12^ ^)^ FGF‐23 is usually decreased,^(^ ^13^ ^)^ however also elevated FGF‐23 levels in TDF‐related hypophosphatemia were observed by others.^(^ ^14^ ^)^

One year before the first contact at our institute, the patient received denosumab due to the fracture history, followed by a low‐traumatic femoral fracture. It has been reported repeatedly that antiresorptive treatment might lead to hypocalcemia and hypophosphatemia in patients with existing osteomalacia. Consequently, denosumab can induce or worsen preexisting hypophosphatemia and osteomalacia in TDF‐acquired Fanconi syndrome, leading to severe bone pain and atraumatic fractures.^(^ ^1^ ^)^ Similar effects were observed after zoledronic acid administration in a patient with undiagnosed TDF‐induced osteomalacia. In this case, a mild hypophosphatemia was already observed before the bisphosphonate administration, with a consecutive aggravation afterward accompanied by several fractures.^(^ ^15^ ^)^ Therefore, antiresorptive agents including denosumab and bisphosphonates must not be administered in patients with untreated osteomalacia or unsolved bone status.

Consistent with the increased ALP, osteomalacia was confirmed by histomorphometry, and hypomineralization was validated by qBEI analyses (Figs. 1, 2, 3). Osteoid volume and thickness were both remarkably increased. The BMDD showed a pronounced shift to lower mineralization values. Interestingly, the shape of the BMDD can well be approximated as the sum of the reference curve from healthy controls and another single peaked curve with reduced mineralization content. This allows the conclusion that 56% of the bone is normally mineralized, while 44% is hypomineralized, indicating a partial remineralization of the bone matrix. This observation is in line with clinical data showing that the patient's phosphate and ALP levels normalized after stopping denosumab treatment and initiating phosphate substitution. Thus, it can be speculated that the BMDD curve shows that 44% of hypomineralized bone material is in the process of mineralization toward normal values. Consequently, the BMDD peak is shifting to the right, merging with the reference curve.

Goldner staining revealed the presence of osteocyte lacunae with peri‐osteocytic lesions (red staining) associated with osteomalacia. Indeed, the OLS evaluation based on quantitative back scattered electron images revealed a higher OLS‐Area and OLS‐Perimeter in both the cortical and trabecular region. Larger osteocyte lacunae have also been reported, in X‐linked hypophosphatemia.^(^ ^10^, ^16^, ^17^ ^)^

Genetical analysis showed a Sp1‐Polymorphism of the COL1A1 gene. This genetic marker was previously suggested as a moderate risk factor for osteoporosis and fractures.^(^ ^18^ ^)^ However, this was not confirmed by others.^(^ ^19^ ^)^

After the final diagnosis of incomplete tenofovir‐induced Fanconi syndrome and osteomalacia, we referred our patient to a clinic specializing in treatment of infectious diseases. The patient was put on a course of antiviral therapy with tenofovir pro‐drug tenofovir‐alafenamide (TAF) due to an increasing viral load. TAF was reported to have the same efficacy at lower plasma levels but a beneficial safety profile compared with TDF. It is currently unclear if the switch from TDF to TAF results in a full resolution of Fanconi syndrome in affected patients. However, an improvement of renal tubulopathy has been previously reported in an HIV‐ and hepatitis B‐coinfected patient.^(^ ^20^ ^)^ Nevertheless, TDF treatment discontinuation seems to be the most important therapeutic step in TDF‐induced osteomalacia. Despite an improvement in muscle strength and bone pain, a normalization of laboratory results was observed 6 months after the cessation of TDF in a patient with incomplete Fanconi syndrome.^(^ ^13^ ^)^

**Fig. 5:**
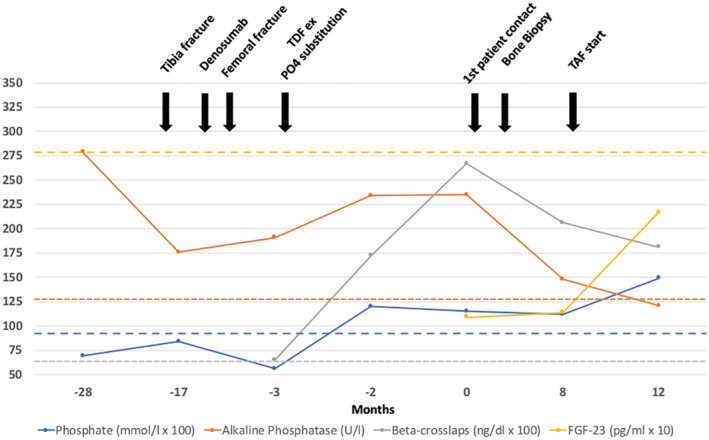
Changes in phosphate, alkaline phosphatase, beta‐crosslaps (CTX), and FGF‐23 levels at different time points relative to the admission to the outpatient clinic. The most important therapeutic and clinical landmarks are shown in the graph. For purposes of graph readability, the original phosphate and beta‐crosslaps values were magnified by a factor of 100, while the FGF‐23 level was magnified by a factor of 10. Yellow dotted line = lower limit of normal (LLN) for FGF‐23; orange dotted line = upper limit of normal (ULN) for alkaline phosphatase; blue dotted line = LLN for phosphate; gray dotted line = ULN (stratified by sex and age) for crosslaps (0.150–0.635 ng/mL) taken from Jorgensen and colleagues.^(^ ^21^ ^)^

Our patient presented with normal kidney function but proteinuria and glucosuria as well as remarkably increased ALP and CTX values. After cessation of TDF, phosphate and alkaline phosphatase levels returned to normal and CTX decreased. The patient continued receiving oral phosphate and 25‐OH‐vitamin D substitution. She sustained one new metatarsal fracture after TDF therapy was withdrawn; however, in general she made a significant recovery up to this point in time. A chart summarizing the therapeutic and clinical milestones of the patient's recovery is shown in Fig. 5.

TDF is a known risk factor for low‐trauma fractures due to different effects on bone metabolism. This case report highlights the importance of a detailed investigation of the cause of bone disturbance and therefore provides the correct treatment for these patients. For the diagnosis of TDF‐induced Fanconi syndrome and consecutive osteomalacia, blood and urine examinations are the first indicators. In addition, bone scintigraphy might be a helpful tool to detect increased uptake due to pseudofractures.^(^ ^22^ ^)^ However, a precise diagnosis should include a transiliac bone biopsy to assure the diagnosis of osteomalacia with histomorphometry.

## Disclosures

All authors state that they have no conflicts of interest.

## Authors' roles:

All of the authors have contributed equally to the creation of the first draft, furher development process and were likewise involved in the process of review‐writing and editing. Jochen Zwerina, MD performed the careful supervision of the project together with Roland Kocijan, MD whose role is also that of the corresponding author.

## Author contributions

**Benjamin Hadzimuratovic:** Conceptualization; data curation; formal analysis; investigation; methodology; project administration; resources; visualization; writing‐original draft; writing‐review & editing. **Judith Haschka:** Conceptualization; data curation; formal analysis; investigation; resources; visualization; writing‐original draft; writing‐review & editing. **Markus Hartmann:** Conceptualization; data curation; formal analysis; investigation; resources; visualization; writing‐original draft; writing‐review & editing. **Stéphane Blouin:** Conceptualization; data curation; formal analysis; investigation; resources; visualization; writing‐original draft; writing‐review & editing. **Nadja Fratzl‐Zelman:** Conceptualization; data curation; formal analysis; investigation; resources; visualization; writing‐original draft; writing‐review & editing. **Jochen Zwerina:** Conceptualization; project administration; supervision; writing‐review & editing. **Roland Kocijan:** Conceptualization; data curation; formal analysis; investigation; methodology; project administration; resources; supervision; visualization; writing‐original draft; writing‐review & editing.
